# Binge drinking and insomnia in students from health sciences at one university in Rio de Janeiro, Brazil

**DOI:** 10.1590/1414-431X202010679

**Published:** 2021-05-24

**Authors:** V.A. da Silva-Fonseca, F.B. Vásquez, A. Seixas, G. Jean-Louis, M.S. da Silva-Fonseca, L. Sladek, E.M.S. da Rocha, R.M.M. Santos, A.S. de Aguiar

**Affiliations:** 1 Núcleo de Ciências Comportamentais e do Desenvolvimento, Instituto Biomédico, Universidade Federal Fluminense, Niterói, RJ, Brasil; 2 Programa de Pós Graduação em Saúde Coletiva, Universidade Federal Fluminense, Niterói, RJ, Brasil; 3 Center for Healthful Behavior Change, Department of Population Health, NYU School of Medicine, New York, NY, USA; 4 Departamento de Nutrição, Universidade Federal de Juiz de Fora, Minas Gerais, MG, Brasil

**Keywords:** Sleep, Alcohol, University, Insomnia, Binge drinking, Sex

## Abstract

In spite of the many studies examining alcohol consumption, recent reviews have indicated that binge drinking has not been extensively studied. Furthermore, it is becoming increasingly clear that sleep is associated with many physiological functions and to drug addictions. The present study aimed to evaluate the relationship between alcohol binge drinking and insomnia in college students of health sciences. All first-year health sciences students (n=286) were evaluated in a cross-sectional study. Envelopes containing the Insomnia Severity Index (ISI), the Alcohol, Smoking, and Substance Involvement Screening Test (ASSIST), and questions capturing sociodemographic data were distributed and collected in classes. It was found that most non-drinkers were female (70.6%), although there were no sex-related differences in the number of binge drinkers (more than 5 drinks on each occasion at least once a week), allowing statistical comparison. The Mann-Whitney U test indicated that the ISI scores were significantly greater in female than male binge drinkers (P=0.014). Moderate or severe insomnia was reported by 23% of the sample, with alcohol being the most frequently associated substance. A specialized intervention was suggested by ASSIST: brief for marijuana (19.2%) and tobacco (23.3%) use, and moderate (31.5%) or intensive (1.4%) for alcohol consumers. The data highlighted the need to pay attention to the habits of college students beyond obtaining scientific information. New data suggesting the influence of genetics on insomnia may be of importance when performing additional studies on the sex differences in alcohol binge drinking.

## Introduction

The National Institutes of Health defines binge drinking in adulthood as the consumption of 5 or more alcoholic drinks in about 2 h (1 drink=10 g of alcohol) (1). Further, alcohol binge drinking is recognized as a major contributor to the social and health burdens associated with alcohol consumption. Alcohol binge drinking is associated with fights, accidents, suicidal ideation, blackouts, and insomnia (2). Insomnia is the second-most prevalent mental disorder, with no efficient treatment available; recent research has also suggested there may be an element of substantial heritability (3). The underlying pathophysiological processes and causal relationships between insomnia and disease are poorly understood, but there may be a genetic relationship, as 57 loci for self-reported symptoms were identified (4). In addition, a causal link has been found between insomnia and coronary artery disease, depressive symptoms, and subjective well-being, all of which may be genetically related (4). An interaction between social distress and genetic predisposition is possible, as epigenetics are often found when psychiatric disorders are studied. The relationship between - and the ability to predict the onset of - insomnia and several mental disorders is of importance. Research employing sleep monitoring strategies, either using actigraphy or band wrists with a specific application, has been useful for detecting anxiety and predicting mood disorder relapse (5). It was also reported that detected sleep disturbances can predict increased cravings for substances such as cocaine, even before drug relapse, which is of great interest both for treatment and public health (6). There is scant information available on this subject.

There is also a dearth of research available on alcohol misuse relapse, and there is even less available on the sex differences related to sleep disturbances after alcohol intoxication and withdrawal. A sex-dependent effect has been reported in insomnia, as it was more frequently found in female drinkers than in male drinkers (7,8); however, the daily activities of the subjects were not reported and there were wide age intervals. However, important studies related to sex differences were carried out to explore the effects of other drugs on sleep in adolescents. For instance, sex differences in sleep patterns have been investigated, and they were evident in caffeine-related parasomnias (9). In a nationally representative sample, one study explored the relationship between alcohol use and sleep problems in adolescents and young adults. Sex differences in the associated consequences of binge drinking were described (10).

Although important studies have been carried out to explore the relationship between sleep and alcohol consumption in adolescents, studies exploring the same relationship in college students from Brazil, especially those in health sciences, have not been published to our knowledge. This is especially true for studies that investigate the relationship between the consumption of other drugs and the degree of severity of insomnia. Binge drinking is a common pattern in university students and a matter of concern, as alcohol-based parties are extensively advertised. Holding alcohol-based freshman-year parties in universities is an old habit, and these parties still exist. As federal universities receive students from across the country, most of these students are usually living alone for the first time in their lives, having moved out from their family homes (11). In fact, most of these students live in groups, sharing accommodations and lifestyles (11). In young university students, factors including distance from the family, life independence, new friends, and distance of old friends have been studied and were found to be related to loneliness. The age of exposure to these life events coincides with the time at which students are most susceptible to experiencing an initial outburst of many psychiatric disorders and substance dependence (12). Studies exploring medical students' alcohol habits have shown that learning about the dangers of alcohol intake during their medical education was not associated with changes in drinking patterns (13). These data indicate that scientific information is not enough to protect against social factors.

The main objectives of the present study were 1) to evaluate the prevalence of self-reported insomnia among college students; 2) to evaluate whether students in health sciences require intervention for drug consumption; and 3) to determine the level of intensity of an intervention on consumption of alcohol and other drugs based on the relationship between alcohol binge drinking and insomnia symptoms.

## Material and Methods

The study was approved by the Research Ethics Committee at the Federal University of the State of Rio de Janeiro, Niteroi, RJ, Brazil (UFF; CAAE code: 98435018.9.0000.5243). Written informed consent was obtained from all participants.

### Sample

First-year college students enrolled in health sciences (any field) from the UFF were recruited to take part in this study. University admissions occur through a national selection process, to which students from all Brazilian states can apply. All students enrolled in the health sciences program were invited to participate. To avoid the potential embarrassment associated with declining to participate, the students had the option to return unfilled questionnaires to the research team in a closed and anonymous envelope. As there was only one opportunity to receive the questionnaires, students who were absent from the classes we visited were not contacted. All students who are enrolled in a health sciences program tend to share classes at the same Biomedical Institute during their first year at the university. In the present study, the health areas investigated were medicine, veterinary medicine, nutrition, psychology, biomedicine, and nursing, given that health encompasses the health of society and individuals.

### Procedures

Professors were contacted prior to study implementation, and the optimal day and time for data collection were established. Students did not receive communication about this process. The students were recruited in class, and a member of our team distributed closed envelopes that separately contained the informed consent form and study questionnaires. Students were then asked to complete the questionnaires if they chose to participate; both complete and unfilled questionnaires were collected in closed envelopes. The total sample was comprised of 286 students. The collected questionnaires were verified and incomplete questionnaires were discarded. Anonymity was maintained throughout the entire research process.

### Instruments

#### Insomnia Severity Index (ISI)

The ISI questionnaire features validated cutoff points to classify insomnia as light, moderate, or severe. It can also be used to assess the total insomnia score; in this case, higher scores are indicative of more intense insomnia. The scale consists of 7 statements that are answered using Likert-type responses. The response of “I find my sleep unsatisfactory” is typically the most frequently selected statement. The validated Portuguese version of the ISI was used (12).

#### Alcohol, Smoking, and Substance Involvement Screening Test (ASSIST)

This measure was elaborated by the World Health Organization (WHO) and was translated and validated in Portuguese (13). This version of the instrument was employed to measure the use of most psychotropic drugs, including sedatives and hypnotics. It is a very useful tool when aiming to understand the drug use habits of various populations, as it provides guidance on the intensity of necessary preventive or therapeutic interventions for each drug. As ASSIST does not rate the amount of drug ingested on each occasion, our team added a question to explore the quantity of alcohol ingested on each occasion for the purposes of this research.

#### Sociodemographic data

A questionnaire inquiring about participants' age, sex, sexual orientation, previous and current diseases, and current medications was included. For the purpose of our analysis, only sex - and not sexual orientation - was considered.

### Statistical analysis

The Statistical Package for the Social Sciences (SPSS) v. 23.0 (IBM Corporation, USA) was used for parametric or non-parametric analysis to explore data normality, homoscedasticity, and continuity.

## Results

### Descriptive results

There were 286 volunteers in the entire sample. However, because of incomplete or unclear responses, the responses of 216 participants were analyzed across all variables. Of the respondents who participated, 144 were female, 71 were male, and 1 did not declare. The median age was 20 years, with no significant differences between sexes. No important reports of diseases or medication were noted. Although most of the non-drinkers were female (70.6%), the number of those presenting with binge drinking patterns (i.e., consuming more than 5 drinks in one occasion, and at least once a week) did not differ between sexes. This made it possible to compare the samples without potential bias.

Moderate or severe insomnia was reported by 23% of the sample. Furthermore, alcohol was the most prevalent drug among all the drugs examined in this population. Specialized intervention was suggested by ASSIST, where brief intervention was suggested for marijuana (19.2%) and tobacco (23.3%) use, and moderate (31.5%) or intensive (1.4%) intervention was recommended for alcohol consumers.

### Inferential results

An interesting finding emerged comparing binge-drinking females and non-drinking females in terms of the presence of moderate and severe insomnia (Table 1). Although not a primary objective of the study, the data suggested that there were notable sex differences in terms of insomnia. Therefore, the sexes were analyzed separately, where male and female binge drinkers were compared, as there were similar numbers of participants in both groups. Female binge drinkers had a significantly higher ISI score. This difference was not evident among males. As the number of binge drinkers did not differ between sexes, a comparison between the total ISI scores for female and male binge drinkers was made to confirm the differences noted in alcohol consumption without sample size bias. Female binge drinkers had significantly higher ISI scores than did male binge drinkers, according to the results of the Mann-Whitney U test (P<0.014; Table 1).

**Table 1:** Insomnia Severity Index (ISI) total scores in non-drinkers and binge drinkers by sex.

Sex	Non-drinkers	Binge drinkers
Female (n=129)	10.5 (0-23) (n=36)	14.0 (5-24)* (n=24)
Male (n=62)	7.0 (4-15) (n=13)	8.5 (4-22) (n=22)

Data are reported as median (minimum-maximum). *P=0.014, binge-drinking females *vs* non-drinking females (Mann-Whitney U test).

The ASSIST scores indicated that alcohol was the main drug requiring moderate to intense intervention. However, other drugs were also considered for intervention, including tobacco, which has been the focus of intensive public health prevention measures (14).

## Discussion

Alcohol consumption in universities across Brazil is considered an important problem, with binge drinking representing the most common consumption pattern (15). Other studies by our group that have focused on medical students demonstrated that alcohol consumption does not change throughout the students' progression in the medical program, thus suggesting that information about the dangers of alcohol intake is not enough, and that preventive measures should be taken when necessary (13). The data from this study indicated that there is a significant relationship between insomnia and binge-drinking patterns, but only in females. We also observed that there was a significant need for drug consumption intervention. As the sample in the present study was comprised of college students whose professors were health professionals, knowledge of the need for intervention to address alcohol and other drug consumption was ethically mandatory.

It is not surprising that females were more susceptible to the toxic effects of alcohol, as many studies have reported this outcome (16). It is known that women drink less, as was reconfirmed in the present study, and it has also been reported that those who drink more develop medical problems and generally experience worse effects associated with alcohol toxicity (13). Conversely, as far as we know, studies on alcohol-related insomnia have not identified whether self-reported insomnia persists for days after intoxication. There are many studies that have explored the effects of ethanol on self-reported sleep; however, the results have differed between sexes across different ethnic populations (17).

The acute effects of binge alcohol drinking on sleep, as well as the general effects of chronic heavy drinking, are well known. Alcohol is a central nervous system depressor and interacts with several neurotransmitter systems that are important in sleep regulation. Most of alcohol's effects are related to the substance's action on GABA receptors, as well as to its antagonizing effects on glutamate NMDA receptors (18). The acute consumption of large amounts of alcohol prior to sleep leads to decreased sleep-onset latency and changes in sleep architecture early in the night, when blood alcohol levels are high. This subsequently results in disrupted, poor-quality sleep later in the night. The effects of alcohol withdrawal on sleep have also been described (19).

The present study adds to the currently available information on alcohol toxicity. Insomnia among university students in health-related programs requires further study. The number of male and female binge drinkers was the same; as such, no sample size-related biases were expected. It is thus possible to conclude that female students are more susceptible than are male students to the effects of binge drinking on sleep. This study also pointed out the need for intervention to address drinking habits, although interventions for the use of other drugs should not be forgotten.

Additional studies are needed to clarify the duration of the effects of binge drinking on sleep, and if university-based prevention strategies could be effective in this particular situation.

### Study limitations

The present study has the following limitations: only students present in class on the day of evaluation were included. It is thus possible that those most affected by binge drinking or associated insomnias were not present. Further, a larger study sample is needed to allow for other appropriate statistical analysis approaches to be conducted, such as binary logistic regression. The number of non-responders in this study was small, but their characteristics were not analyzed. Finally, this study included a subset of students from the university, representing a convenience sample. A more comprehensive study is currently underway and is being carried out in collaboration with the epidemiology department at the Collective Health Institute. Although the primary research questions were answered, much research remains to be conducted in this field.
